# Drug-eluting Microspheres Compared to Conventional Transarterial Chemoembolization as First Line Treatment for Unresectable Hepatocellular Carcinoma: A Single-center Retrospective Cost-utility Analysis

**DOI:** 10.1007/s00270-022-03335-4

**Published:** 2023-01-04

**Authors:** G. Andreozzi, V. Lorenzoni, I. Bargellini, R. Cioni, G. Turchetti

**Affiliations:** 1 Institute of Management, Scuola Superiore Sant’Anna, Piazza Martiri Della Libertà, 33, 56127 Pisa, Italy; 2 Department of Interventional Radiology, Pisa University Hospital, Pisa, Italy

**Keywords:** TACE, Drug-eluting microspheres, Economic, Cost, Cost-utility

## Abstract

**Purpose:**

To assess the cost-utility of initial treatment with drug-eluting microspheres (DEM) transarterial chemoembolization (TACE) versus conventional (C)-TACE in patients with hepatocellular carcinoma considering the perspective of a Local Healthcare Authority in Italy.

**Materials and Methods:**

The economic evaluation is based on a retrospective single-center study and individual patients’ data whose details have been previously reported. The impact of initial treatment with DEM-TACE or C-TACE on disease progression, mortality, and direct health costs over a lifetime horizon were simulated and compared in terms of incremental cost-utility ratio expressed as costs per quality adjusted life years (QALY). Costs included direct health costs related to the first chemoembolization procedure and all subsequent follow-up costs associated with health care resources used for disease management. Probabilistic (PSA) sensitivity analysis was used to assess the robustness of the results.

**Results:**

A total of 101 patients in each treatment group were considered. All over the time-horizon median costs were €3,145.14 and €2,158.32 in the DEM-TACE and C-TACE group, respectively (*p *< 0.001); while mean costs were € 24,619 and € 17,001, respectively (*p *< 0.001). The ICUR was 6,461.86 €/QALY when using median costs derived from the study population as input for the health-economic evaluation and 49,932.15 €/QALY when the mean costs were considered. Results from PSA highlighted that using median costs DEM-TACE was always cost-effective, while using mean costs, it was preferable only 24.7% of times.

**Conclusions:**

The higher prices of DEMs are counterbalanced by the positive impact on QALY.

**Supplementary Information:**

The online version contains supplementary material available at 10.1007/s00270-022-03335-4.

## Introduction

Hepatocellular carcinoma (HCC) is the most frequent malignant liver cancer [1], largely triggered by liver cirrhosis [2, 3] and prognosis of HCC patients mainly depends on the stage at which the disease is diagnosed, where staging is based not only on tumor extent but also on the patient's clinical condition and liver function [3]. Although there are potentially curative treatments (resection, liver transplantation and percutaneous ablation), many HCC patients are still diagnosed at a stage where only palliative treatments are indicated.

Transarterial chemoembolization (TACE) is the treatment of choice in patients excluded from curative treatment [4–6], and it frequently represents the first-line treatment across different stages being recommended by guidelines for both early and the intermediate stage [7–10].

To date, however, TACE includes several transarterial therapeutic modalities that differ in the types of drugs administered and arterial embolization modalities without clear recommendation for the use of the diverse approach, in the absence of clear evidence of superiority of one technique over another [11].

In the last decade, a new technology has been developed for TACE represented by embolizing particles capable of absorbing the chemotherapy and selectively releasing it into the neoplastic tissue over time (drug-eluting microspheres, DEM). Compared to conventional (C-) TACE, which involves intra-arterial administration of chemotherapy mixed with Lipiodol® accompanied by subsequent arterial embolization with permanent embolizing particles or resorbable material, DEM-TACE would be able to increase the intratumoral concentration of chemotherapy and reduce its release into the systemic circulation, with the possibility of implementing intratumoral efficacy while reducing liver and systemic toxicity of the treatment.

Considering the health-economics perspective, available evidence based on retrospective data or on models developed on the basis of literature evidence suggests a favorable pharmacoeconomic profile for DEM-TACE because of lower rate of hospitalization, adverse event and associated costs due to the reduced toxicity of DEM-TACE compared to C-TACE [12, 13].

Further studies based on real clinical practice rather than on randomized controlled trials, capturing the spectrum of all costs and consequences in the peri-procedural and post-procedural period over a lifetime horizon and also accounting for real costs of procedures are needed to shed light on the clinico-economic implications of an alternative approach to guide treatment decision. Accounting for patients’ preference is also advisable.

Accordingly, the present study aims at performing a cost-utility analysis to compare initial treatment with DEM-TACE versus C-TACE on the basis of data from a single-center retrospective study, estimating real costs of TACE procedure and also accounting for follow-up costs. The economic evaluation is performed considering a Markov model depicting disease progression (considering the target lesion) and adopting the perspective of the Local Healthcare System (LHS).

### Methods

The present study aims at evaluating the economic impact of first treatment with either DEM or C-TACE considering individual patients’ data whose details have been previously reported [14].

In details, a cost-utility analysis considering the perspective of the LHS, thus accounting only for direct health costs, was performed to compare initial treatment with DEM-TACE and C-TACE in HCC patients over a lifetime horizon. The analysis was based on a Markov model developed on the basis of single-center individual patient data retrospectively collected and allowed evaluating incremental costs-utility ratio (ICUR), expressed as incremental costs per Quality Adjusted Life Years (QALY), of DEM-TACE vs C-TACE.

Moreover, given the skewed distribution of costs data, analyses were performed assuming costs for the different health states based on (1) median costs or (2) mean costs derived from the study population with the intention to offer the range of possible real economic impact of the approaches compared.

### Population and Data Collection

Briefly, as extensively described previously [14], individual patient data about naïve patients with unresectable HCC undergoing initial treatment with either DEM-TACE or C-TACE between January 2006 and December 2013 were retrospectively extracted from clinical charts of the Department Interventional Radiology in a large hospital of central Italy; similarly their follow-up data (i.e., treatments, clinical events and resources use) until death or the end of follow-up, July 2019, were retrieved.

Patients undergoing either DEM-TACE or C-TACE were 1:1 matched using a propensity score approach and considering a logistic model in which baseline Barcelona Clinic for Liver Cancer (BCLC) stage, uninodular or multinodular tumor and tumor extension (unilobar or bilobar) were used as matching variables.

### Structure of the Model for Cost-utility Analysis

A Markov model accounting for the perspective of a LHS in Italy was developed to estimate and simulate the impact of initial treatment with TACE on disease progression (considering the target lesion), mortality and direct health costs.

In particular, the Markov model mimics disease progression (considering the target lesion) accounting for three health states: stable disease, disease recurrence and death. Event-free survival and recurrence-free survival were estimated from individual patients’ data by means of survival analysis as detailed in Supplemental online material, and transition probabilities between health states were derived considering the Aalen-Johansen estimator.

**Fig. 1:**
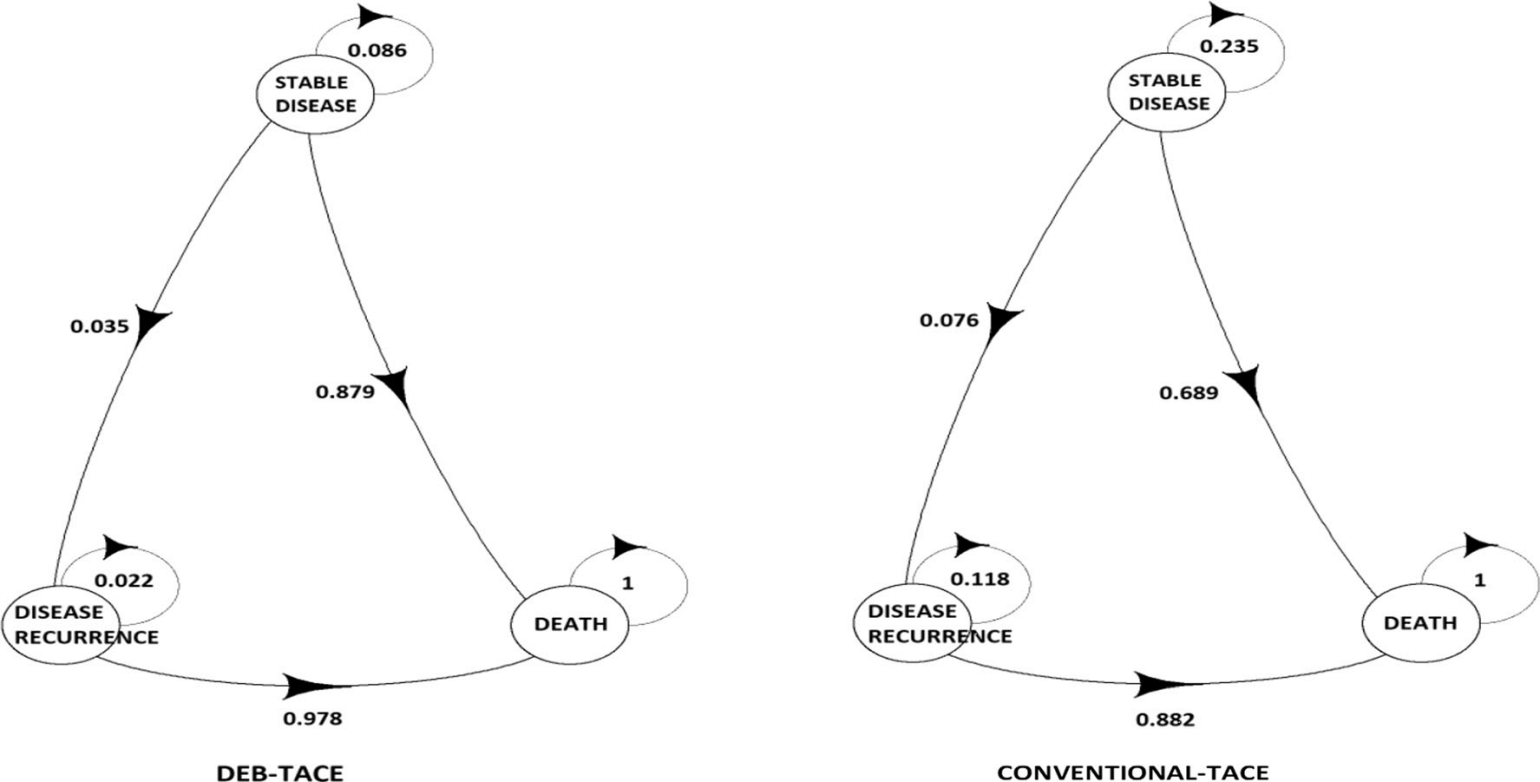
Schematic representation of the Markov model used in the analysis. The figure provides a representation of the Markov model used in the analysis for each treatment arm. Three different health states were considered in the model and they are represented in figure by a circle, arrows between them represent transition allowed in the model and numbers superimposed correspond to values of the transition probabilities used in the base case analysis

Figure 1 depicts model scheme and transition probabilities considered in the base case analysis.

The model developed allowed estimation and comparison of overall direct health costs related to the initial TACE procedure, subsequent treatments and events as well as QALY of the DEM-TACE and C-TACE group over a lifetime horizon and considering 1-year cycles.

### Utilities and Costs

On the basis of event-free survival and recurrence-free survival estimated from individual data and utility values associated with each state derived from literature [15], QALY were estimated considering time spent in the different health states. Utility values used in the analysis are listed in Supplemental online material. Despite transplant was not considered as a separate health state in the model, the utility value related to transplant was considered when deriving QALY associated to the different health state as values assigned to each health states were weighted to account for the proportion of patients undergoing or not transplant.

Direct health costs considered in the analysis comprised costs associated with the chemoembolization procedure, drug use, length of hospital stay, subsequent treatments, instrumental and imaging tests as well as hospitalizations and procedures performed during the follow-up for disease-related events (i.e., recurrence).

All costs were expressed in Euro2020 and valued according to official administrative sources available from the local health authority or at national level [16, 17]. Details of costs used in the analysis are provided as Supplemental online material.

### Sensitivity Analysis

A probabilistic sensitivity analysis (PSA) was performed to assess robustness of results obtained from the base case analysis. Appropriate probability distributions were assigned to all parameters (see Supplemental online material) and 10,000 simulations were performed drawing samples simultaneously from those distributions to evaluate uncertainty. Results from the PSA were represented on the cost-effectiveness plane.

## Results

### Characteristics of the Study Population

As already detailed in a previously published clinical paper [14], out of an initial series of 656 naïve HCC patients who underwent TACE from January 2006 to December 2013, 101 patients in each treatment group were retained in the present analysis, after the propensity score matching. Main clinical characteristics were homogeneous in the two groups, with the exception of the administered doxorubicin dose, which was significantly higher in the DEM-TACE group. A greater number of subjects in the C-TACE group had disease recurrence compared to the DEM-TACE group (62 and 49, respectively) [14].

### Effectiveness and Costs

A total of 62 patients (61.4%) experienced disease recurrence in the C-TACE group over the follow-up time, in the DEM-TACE group recurrence occurred in 49 (48.5%) patients (*p *= 0.202). The number of death observed was 77 (76.2%) and 58 (57.4%) in the C-TACE and DEM-TACE group, respectively (*p *= 0.007).

Procedural costs were similar in the two groups as the higher costs of consumables in the DEM-TACE group were offset by reduced hospitalization in this groups (mean length of stay being, respectively, 1.9 vs 2.5 days, *p *= 0.030) and thus lower costs associated with the length of stays.

Table 1 shows median and mean costs estimated and associated with either stable disease or disease recurrence.

**Table 1:** Costs related to stable disease and recurrence in the conventional and DEM-TACE groups

Health state	Stable disease		*p*	Disease recurrence		*p*
	C-TACE	DEM-TACE		C-TACE	DEM-TACE	
Costs (€), Median [IQR]	0 [4085]	0 [4085]	0.882	4415 [11,067]	4085 [16,340]	0.361
Costs (€), Mean ± Standard Deviation	2463 ± 6626	2158 ± 5067	0.714	12,406 ± 17,361	11,972 ± 17,156	0.895

*C-TACE* conventional transarterial chemoembolization, *DEM-TACE* drug-eluting microspheres transarterial chemoembolization, *IQR* interquartile range

### Health-Economic Evaluation

Considering median costs derived from the study population to represent costs associated with the different health states, median costs per patient estimated through the Markov model all over the lifetime horizon were €3,145.14 and €2,158.32 in the DEM-TACE and C-TACE group (*p *< 0.001), respectively. Cost-utility ratio (cost/QALY) was estimated to be 6,009.30 €/QALY for the DEM-TACE treatment and 5,823.11 €/QALY for the C-TACE treatment. The ICUR was calculated to be 6,461.86 €/QALY (Table 2).

**Table 2:** Results from the cost-utility analyses performed

	Cost-utility analysis based on median costs	Cost-utility analysis based on mean costs				
	DEM-TACE	C-TACE	*p*	DEM-TACE	C-TACE	*p*
Cost	€ 3,145	€ 2,158	<0.001	€ 24,619	€ 17,001	<0.001
QALY	0.523	0.371	<0.001	0.523	0.371	<0.001
Cost/QALY	6,009	5,823	<0.001	47,039	45,849	<0.001
ICUR (€/QALY)	6,462			49,932		

*C-TACE* conventional transarterial chemoembolization, *DEM-TACE* drug-eluting microspheres transarterial chemoembolization, *ICUR* incremental cost-utility ratio, *QALY* quality adjusted life years

When considering mean costs to represent costs associated with the diverse health states, overall mean costs per patient estimated through the Markov model over the lifetime horizon were €24,619.13 and €17,001.55 in the DEM-TACE and C-TACE group (*p *< 0.001), respectively. The cost-utility ratio (cost/QALY) was estimated to be 47,038.92 €/QALY for the DEM-TACE treatment and 45,848.62 €/QALY for the C-TACE treatment. The ICUR was 49,932.15 €/QALY (see Table 2).

In both cases, QALYs were estimated to be 0.452 for the DEM-TACE treatment and 0.318 for the C-TACE treatment.

### Sensitivity Analysis

To investigate model structural uncertainty, a probabilistic sensitivity analysis (PSA) was performed assigning different distributions for each of the inputs used.

In details, normal distributions were used for costs (as we used tariffs for almost all the costs modeled), beta distributions were used for QALYs and Dirichlet distributions were used for the transition probabilities.

**Fig. 2:**
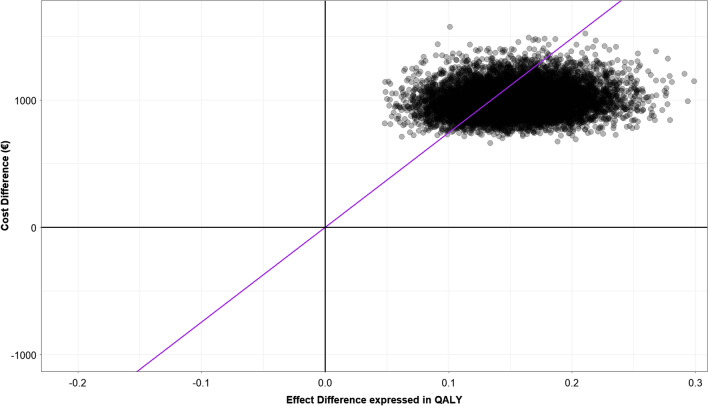
Results from probabilistic sensitivity analysis when median costs are used as input in the model. The cloud represents results from the probabilistic sensitivity analysis when median costs are used as input in the model displayed in the cost-effectiveness plane along with the line representing a willingness to pay (WTP) of €25,000/QALY. The cloud representing results from the 10,000 simulations performed are consistently located in the first quadrant, meaning that the results of the analysis were consistent

**Fig. 3:**
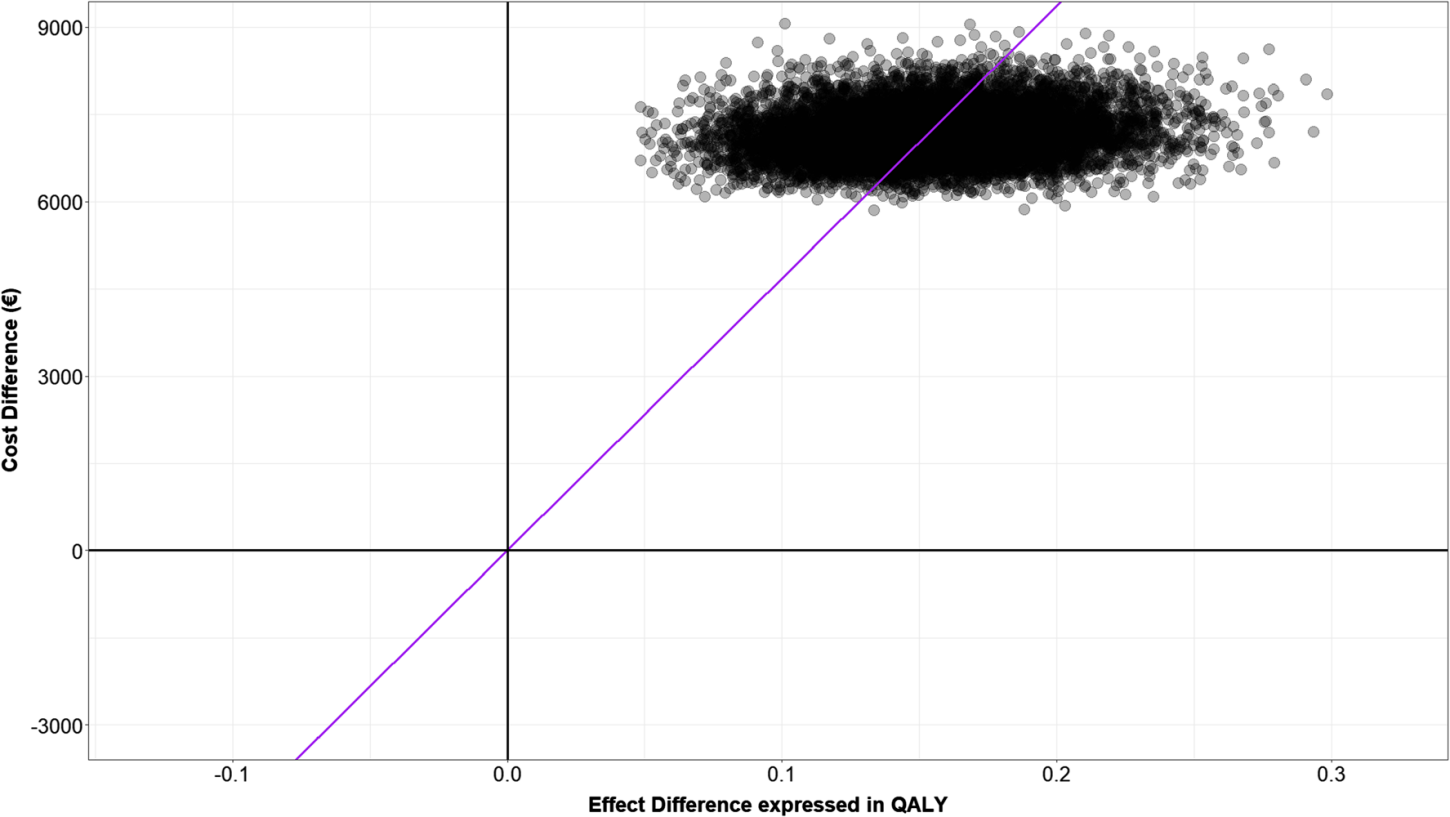
Results from probabilistic sensitivity analysis when mean costs are used as input in the model. The cloud represents results from the probabilistic sensitivity analysis when mean costs are used as input in the model displayed in the cost-effectiveness plane along with the line representing a willingness to pay (WTP) of €25,000/QALY. The cloud representing results from the 10,000 simulations performed is consistently located in the first quadrant, meaning that the results of the analysis were consistent

Figures 2 and 3 depict results from the PSA in the cost-effectiveness plane for the two scenarios considered in the present analysis and considering 10,000 simulations.

In both cases, PSA suggests that results from the analysis performed were consistent as points simulated were all located in the first quadrant of the cost-effectiveness plane. In details, when considering median costs to represent costs associated with the diverse health states, DEM-TACE was always cost-effective using a threshold of €25,000/QALY; while using mean costs to depict costs related to the diverse health states, DEM-TACE was preferable only 24.7% of times.

## Discussion

Results from the analyses conducted showed that DEM-TACE and C-TACE produced similar impact on the cost side while DEM-TACE was associated with a slightly increase in QALY thus delivering potential benefit to patients.

When evaluating the health-economic implications, based on the analyses performed (considering both median and mean costs estimated from individual patient data), results suggested that the value for money was comprised between 6,461.86 €/QALY and 49,932.15 €/QALY; despite no official willingness to pay (WTP) threshold exists in Italy, considering well-accepted threshold from other EU countries, typically comprised between €25,000–40,000/QALY, [18, 19] results from the present analyses suggested that while ICUR obtained considering median costs is acceptable, on the other hand, when assuming mean costs results are slightly above the WTP threshold. In particular, despite no significant differences in life expectancy, the slightly higher values found for the DEM-TACE group together with the high cost of transplant that enables healing determined slightly higher values for the median and mean cost in that group.

The limits for the ICUR values obtained considering in the Markov model median and mean costs from individual patient data and reasons for the large difference found between mean and median values of costs estimated for the diverse health states are mainly related to the high costs of liver transplant that was performed in 18.3% of the patients considered in the present analysis. Of notice, a higher number of patients were transplanted in the DEM-TACE group (23, 22.8%) compared to the C-TACE group (14, 13.9%), thus explaining the higher mean costs observed.

DEMs are relatively novel and costly devices, whose clinico-economic profile has been poorly evaluated. The few studies available about the health-economic implication of DEM-TACE showed results that are somewhat in line with our findings [12, 13]. In details, a real-life study conducted in France [12] compared different time periods, with and without the possibility of using DEMs, without however specifically comparing the different TACE techniques. They found no differences between the two time periods in terms of effectiveness, while underlying a significant reduction in hospitalizations for TACE-related toxicities that resulted in a better health-economics profile with the introduction of DEMs in clinical practice.

Another study performed a cost-effectiveness analysis of DEM- versus C-TACE, summarizing with a meta-analysis available evidence about the effectiveness of TACE the study found a significant improvement of QALY for DEM-TACE at a price of a slight increase in costs thus resulting in high probability of DEM-TACE being cost-effective even for willingness to pay of about 4,000€/QALY [13].

Previous studies also suggested the discrepancies between DRG set for DEM-TACE and its real costs [20], thus highlighting the need of adequate reimbursement related to the provision of services implying the use of newer expensive technology to not limit their use.

Compared to C-TACE, DEM-TACE is capable of slow release of the anticancer agent and this may contribute to better tolerability and reduced hospitalization with comparable procedural costs [20–22]; moreover, in patients achieving complete radiological tumor response at one month, DEM-TACE is associated with more durable target tumor response [17]. As in previous studies [23–27], no differences were found with respect to major clinical outcomes, anyway the characteristics of DEM-TACE, also comprising the significantly lower number of treatment, make it more appealing [26] with possible positive impact on patient quality of life (QoL), and this could justify the need to produce more evidence to shed the light about the most effective and sustainable option for HCC.

Main limitation of the present study relies on the retrospective nature of data on which the economic evaluation has been based on. Particularly, the size of the study sample was limited by availability of medical charts. Being an observational study, a propensity score matching was used to limit possible bias related to treatment allocation based on clinicians’ preference and judgment. No specific subgroups analyses were possible with the available data. Finally, results and conclusions from the study are not easily generalizable because patients come from a single center and they are not representative of the overall population of patients undergoing TACE in view of the selection of patients performed to enable obtain comparable groups. Moreover some costs are based on specific data collected from the center involved in the analysis. Indeed, transferability of results to other context is not immediate as it depends on costs set in different context, as well as clinical practice.

Despite limitations, the study reports data from a single-center retrospective study concerning real-practice and real costs associated with either C-TACE or DEM-TACE as first-line treatment modality in HCC patients, thus contributing to the paucity of literature available about the health-economic implication of DEM-TACE. The higher prices of DEMs are counterbalanced by the reduced length of hospitalization, the durable response and the positive impact on QoL and do not impact overall the cost-effectiveness of DEM-TACE compared to C-TACE during follow-up. Further prospective analyses would be desirable, possibly investigating on the cost-effectiveness in specific subgroups of HCC patients, including the impact on QoL and also comprehensively accounting for the effect of transplant that may have a strong positive impact on both survival and QoL.

## Supplementary Information

Below is the link to the electronic supplementary material.

Supplementary file1 (DOCX 21 kb)
