# Supplementary material for: Drug-eluting Microspheres Compared to Conventional Transarterial Chemoembolization as First Line Treatment for Unresectable Hepatocellular Carcinoma: A Single-center Retrospective Cost-utility Analysis
Source: Cardiovasc Intervent Radiol. 2023 Jan 4;46(3):319–26. doi: 10.1007/s00270-022-03335-4 (PMC10014672; doi:10.1007/s00270-022-03335-4)
Supplement: Supplementary file 1 — Supplementary file1 (DOCX 21 kb) [file 270_2022_3335_MOESM1_ESM.docx]

**Supplemental online material**

**Utility values associated to transplant and to the each health states (or event) considered in the model**

| Model state | Utility |
| --- | --- |
| Stable Disease | 0.590 |
| Disease Recurrence | 0.445 |
| Transplant | 0.780 |
| Death | 0 |

**Details of random distributions assigned to model input the PSA**

|  | Random variable | Mean | Standard Deviation |
| --- | --- | --- | --- |
| Percutaneous ethanol injection (PEI) | Normal | 140.35 | 46.78 |
| Transarterial chemoembolization (TACE) | Normal | 4,085 | 1,361.67 |
| Selective Internal Radiation Treatment (SIRT) | Normal | 19,020 | 6,340 |
| Angiography | Normal | 3,060 | 1,020 |
| Radiofrequency ablation (RFA) | Normal | 2,453.5 | 817.83 |
| Sorafenib | Normal | 0.14 | 0.05 |
| Regorafenib | Normal | 0.66 | 0.22 |
| Hepatectomy | Normal | 9,558 | 3,186 |
| Farmorubicine | Normal | 0.91 | 0.3 |
| Lipiodol^®^ | Normal | 29.9 | 9.97 |
| Liver transplant | Normal | 62,648 | 20,882.67 |
| Consumables | Normal | 3,824.88 | 1,274.96 |
|  |  | Alpha | Beta |
| QALY – stable disease | Beta | 3.122 | 3 |
| QALY – disease recurrence | Beta | 2.07 | 3 |
|  |  | Alpha | |
| Transition Probabilities – DEM-TACE | Dirichlet | 1 + transition matrix DEM-TACE | |
| Transition Probabilities – Lipiodol^®^-TACE | Dirichlet | 1 + transition matrix C-TACE | |

C-TACE=conventional transarterial chemoembolization; DEM-TACE=drug-eluting microspheres transarterial chemoembolization

**Approaches and methods for the cost-utility analysis**

*Model for the cost utility-analysis*. A costs-utility analysis was performed accounting for direct health costs and QALY over a lifetime horizon.

In details the cost-utility analysis was performed considering a Markov model comprising three different health states to simulate disease progression. In details patients enter the model being in the health state corresponding to *stable disease*, in subsequent cycles they were allowed to remain in that state or move to either the *disease recurrence* state or the *death* state. Patients in the *disease recurrence* state may just remain in that state or die.

*Effectiveness.* Transition probability between health states were estimated from individual patients data considering the Aalen-Johansen estimator, a generalization of Kaplan-Meier estimator well-suited for multistate model, extrapolation of data over a lifetime horizon were performed considering survival regression models and using a log-normal distribution for the event free survival while a Gompertz distribution was used to model recurrence free survival.

Those model were chosen based on their ability to fit the data. Indeed several semi-parametric and parametric models were assessed and the most appropriate models were chosen evaluating the goodness of fit through the Akaike Information Criterion (AIC). Models showing the best fit were retained and used for data extrapolation.

Using data from the survival analysis and utility values for the different health states from literature [14] QALY were estimated for each arm accounting for time spent in the different health states.

Moreover, to account for the effect of transplant, in each health sate the overall QALY was obtained weighting values to account also for the proportion of patients undergoing or not transplantation.

*Costs.* Costs considered in the model include cost of initial treatment with either DEM- or C-TACE and all subsequent costs related to the consumption of healthcare resources for the management of the disease or for disease related events (i.e., recurrence, transplant).

In details, in order to capture real costs for the two TACE approaches (that would results the same if valued according to tariff) costs associated with the first chemoembolization procedure were estimated valuing resources used within the procedure (i.e., consumables, length of stay) and using unit costs from the accounting office of the hospital involved in the study.

Costs of post-TACE procedures and instrumental evaluations during the follow-up were valued using official administrative sources available from the local health authority or from National reimbursement set by the Italian Ministry of Health when not available at local level [15].

Drug costs were valued considering recommended dosage and unit ex-factory costs from the National Agency for Drugs (AIFA) [16], except for Lipiodol^®^ whose unit cost was obtained from the Accounting Office of the hospital, being no more available in Italy at moment of the analysis.

Details of unit costs considered to value resource use are shown in the table below.

**Details inputs for costs considered in the analysis.**

| **Resource type** | **Unit costs (€)** | **Source** |
| --- | --- | --- |
| **Percutaneous ethanol injection (PEI)** | 140.35 | Tariff sets at local health authority |
| **Transarterial chemoembolization (TACE)** | 4,085 | National DRG reimbursement |
| **Selective Internal Radiation Treatment (SIRT)** | 19,020 | National DRG reimbursement |
| **Angiography** | 3,060 | Tariff sets at local health authority |
| **Radiofrequency ablation (RFA)** | 2,453.5 | Tariff sets at local health authority |
| **Sorafenib** | 0.14 | AIFA |
| **Regorafenib** | 0.66 | AIFA |
| **Hepatectomy** | 9,558 | National DRG reimbursement |
| **Farmorubicine** | 0.91 | AIFA |
| **Lipiodol**^®^ | 29.9 | Hospital accounting office |
| **Drug-eluting micro particles** | 1200 | Hospital accounting office |
| **Liver transplant** | 62,648 | National DRG reimbursement |
| **Consumables** | 2,624.88 | Hospital accounting office |

Mean and median per patient costs estimated on the basis of individual patient data were then estimated for the different health states, in details for stable disease and disease recurrence, and entered as input in the model. Costs of the initial procedure were then added to those costs.
